# Promotion of tumor development in prostate cancer by progerin

**DOI:** 10.1186/1475-2867-10-47

**Published:** 2010-11-24

**Authors:** Yong Tang, Yakun Chen, Hongmei Jiang, Daotai Nie

**Affiliations:** 1 Department of Medical Microbiology, Immunology, and Cell Biology, Southern Illinois University School of Medicine and Simmons Cancer Institute, Springfield, IL 62702, USA

## Abstract

Progerin is a truncated form of lamin A. It is identified in patients with Hutchinson-Gilford progeria syndrome (HGPS), a disease characterized by accelerated aging. The contribution of progerin toward aging has been shown to be related to increased DNA damages. Since aging is one major risk factor for carcinogenesis, and genomic instability is a hallmark of malignant cancers, we investigated the expression of progerin in human cancer cells, and whether its expression contributes to carcinogenesis. Using RT-PCR and Western blotting, we detected the expression of progerin in prostate PC-3, DU145 and LNCaP cells at mRNA and protein levels. Ectopic progerin expression did not cause cellular senescence in PC-3 or MCF7 cells. PC-3 cells progerin transfectants were sensitized to DNA damage agent camptothecin (CPT); and persistent DNA damage responses were observed, which might be caused by progerin induced defective DNA damage repair. In addition, progerin transfectants were more tumorigenic *in vivo *than vector control cells. Our study for the first time describes the expression of progerin in a number of human cancer cell lines and its contributory role in tumorigenesis.

## Introduction

Progerin is a truncated form of lamin A, a major constituent of nuclear lamina, in which 50 amino acid residues are deleted near the C-terminus as a result of a point mutation (1824C > T) in the *LMNA *gene [1]. This mutation was identified in the majority (80%) of patients with Hutchinson-Gilford progeria syndrome (HGPS) [2], a disease characterized by an accelerated aging process [3]. The nucleotide substitution at position 1824 (C to T) in exon 11 of the coding sequence does not result in an amino acid change (G608G) but donates a cryptic splice site that leads to a 150 bp nucleotides deletion in exon 11 of prelamin A mRNA [1]. The truncated prelamin A mRNA is translated into a mutant protein named progerin/LAΔ50 with an internal 50 amino acid deletion. The loss of 50 amino acids at the carboxyl terminus of prelamin A compromises its posttranslational maturation by removing the proteolytic cleavage site, which is required by an integral membrane metalloproteinase ZMPSTE24 (FACE-1) mediated deletion of 15 C-terminal amino acids, thus generating a permanently farnesylated progerin/LAΔ50. Zmpste24-deficient mouse embryonic fibroblasts (MEFs), with relatively high level of prelamin A expression, share phenotypic similarities as HGPS fibroblasts [4].

The accumulation of progerin within HGPS patient fibroblasts or prelamin A in Zmpste24-deficient MEFs acts in a dominant negative manner, causing DNA damages and corresponding DNA damage responses such as constant activation of ATM and ATR, phosphorylation of Chk1, Chk2 and p53 [5,6]. Moreover, HGPS patient fibroblasts and Zmpste24-deficient MEFs are more sensitive to DNA-damaging agents and are retarded in the recruitment of DNA damage response proteins like p53 binding protein 1 (53BP1), indicating the existence of defective DNA repair machinery, such as homologous recombination DNA repair, within these cells [7]. Of note, the inability to carry out functional DNA damage repair, especially for double stranded break repair (DSBR), leads to hypersensitivity to DNA-damaging agents and pronounced genomic instability.

Genetic instability is a key feature of the multi-step tumorigenesis. Loss of genomic stability provides mutations in tumor suppressor genes or oncogenes [8,9]. The present study is undertaken to test the hypothesis that progerin is expressed in cancer cells and that the protein may promote tumorigeneis by increasing genomic instability in cancer cells. We investigated the expression of progerin both at the mRNA and protein levels in several cancer cells lines. Overexpression of progerin failed to induce cellular senescence in PC-3 and MCF7 cancer cells but sensitized PC-3 cells toward DNA damage. The tumors derived from PC-3 cells with ectopic progerin expression showed enhanced growth *in vivo*. Our study is the first to demonstrate the existence of progerin within cancer cells and its possible role in tumorigenesis.

## Materials and methods

### Materials

Human prostate cancer cell lines PC-3, DU145 and LNCap, breast cancer cell lines MCF7 and MDA-MB-231, colon carcinoma cell lines SW480, SW620 and HCT116, and mouse embryonic fibroblast cell line NIH-3T3 were purchased from American Type Culture Collection. MCF10A cells (human mammary epithelial cell-line) were kindly gifted by Randolph C. Elble, Ph.D., the Department of Pharmacology at Southern Illinois University School of Medicine. Fetal bovine serum (FBS) and bovine serum (BS) were purchased from Invitrogen. GFP antibody was purchased from Clontech; Lamin A/C (sc-20681) and emerin antibodies were purchased from Santa Cruz biotechnology; anti-phosphorylated histone-H2AX polyclonal antibody was from Trevigen. Phospho-Chk2 (Thr68) was purchased from Cell Signaling Technology, Inc. Two normal tissues (Normal breast (A804144): 41 y; and Normal colon (A605057): 21 y and two tumor tissues (Breast tumor (A810179): 50 y and Colon tumor (A805131): 51 y) total RNA were purchased from BioChain Institute, Inc. GenePORTER^® ^liposome was from Genlantis. The senescent cell staining kit was from Sigma-Aldrich. CometAssay Kit was from Trevigen. pEGFP-lamin A (denoted as LA-pEGFP), Δ50 pEGFP-lamin A (denoted as progerin-pEGFP) or a control empty vector were kindly given by Dr. Tom Misteli, NIH.

### Cell culture and plasmids transfection

PC-3, DU145, LNCaP, MDA-MB-231, SW480, SW620 and HCT116 cells were grown in a RPMI 1640 medium supplemented with 10% FBS and antibiotics-antimycotics (100 units/ml penicillin, 100 μg/ml streptomycin and 250 μg/ml amphotericin B) in a humidified incubator under an atmosphere containing 5% CO_2 _at 37°C. MCF7 and A431 cells were cultured in a Dulbecco's Modified Eagle's Medium (DMEM) with 10% FBS and antibiotics-antimycotics. NIH-3T3 cells were grown in a DMEM with 10% BS and antibiotics-antimycotics. MCF10A cells were cultured in DMEM/F12 medium, supplemented with Horse Serum, EGF, Hydrocortizone, CholeraToxin, Insulin, and Pen/Strep. PC-3, MCF7 or NIH 3T3 growth to 70%-80% confluence were transfected with expressing plasmids using GenePORTER^® ^liposome transfection reagent following manufacturer's instructions. Stable clones were obtained after fluorescence activated cell sorting (FACS) and G418 (0.4-0.8 mg/ml) selection.

### RT-PCR and real-time PCR

The expression of progerin in established human cancer cell lines was evaluated by RT-PCR. Total RNA was extracted from cell cultures with an RNeasy Mini Kit (QIAGEN) and treated with RNase-free DNase. 2 ug of RNA were reverse transcribed using oligo-dT primers by Superscript TM III First-Strand synthesis system (Invitrogen). Primers for simultaneous detection of full length and truncated *LMNA *isoforms were located in exon 9 and exon 12 of *LMNA *(LMNA-RT9F: 5' GTGGAAGGCACAGAACACCT-3'; LMNA-RT12R: 5'-GTGAGGAGGACGCAGGAA-3') as described in [10]. End-point PCR products were separated in 2% agarose gels and stained with ethidium bromide. Primers for specific detection of truncated *LMNA (progerin) *were located across the aberrant splice junction and in exon 12 (LMNA-RTspecM3F: 5' GCGTCAGGAGCCCTGAGC-3'. Three mismatches were introduced in the primer to avoid primer dimmers formation and cross hybridization with the full-length *LMNA *mRNA; LMNA-RTspec12R: 5'-GACGCAGGAAGCCTCCAC-3'). Primers used to amplify all *LMNA *transcripts were located across exon 8-exon 9 splice junction and in exon 9 (LMNA-RTnorm8F: 5'-GGTGGTGACGATCTGGGCT-3'; LMNA-Rtnorm9R: 5'-CCAGTGGAGTTGATGAGAGC-3'). Samples were analyzed in triplicate in three independent experiments. Use of the cryptic splice site in the different cell lines was calculated by normalizing Δ150 LMNA (progerin) RNA levels to the total LMNA RNA levels in each sample. Samples were analyzed in triplicate in three independent experiments. For the real-time quantitative PCR, the reactions were performed as described by Chen et al [11].

### Western blotting

PC-3 cells with stable progerin-pEGFP, LA-pEGFP or pEGFP vector expression were subjected to different treatments. The cell lyses were collected with ice-cold RIPA buffer (Sigma, R0278) in the presence of a protease inhibitor cocktail (Sigma, S8830). Fifty micrograms of protein were fractionated by SDS-PAGE gels in a Bio-Rad Protean II system. After transferring proteins to a PVDF membrane, the membrane was blocked with Odyssey Blocking Buffer from LI-COR Biosciences for 60 min at room temperature and incubated with the primary antibody at appropriate dilutions in Odyssey Blocking Buffer at 4°C overnight. After overnight incubation with appropriate primary antibodies, the membrane was washed (3×) with TBS-T for a total of 15 min, probed with fluorescently-labeled secondary antibody (1:5000) for 50 min at room temperature and washed (3×) with TBS-T for a total of 15 min. The immunoblots were visualized by an Odyssey Infrared Imaging System (LI-COR).

### Immunocytochemistry and Confocal Microscopy

PC-3 cells with stable progerin-pEGFP, LA-pEGFP or pEGFP vector expression were seeded at 0.3 × 10^6^/well into 6-well plates with coverglasses to achieve 70% confluence. Twenty four hours later, the cells were fixed with 3% paraformaldehyde in PBS for 15 min, followed by permeabilization with 0.1% Triton X-100 for 1 min. After blocking in 1% BSA/1 × PBS containing 3% horse serum for 30 min, the slides were incubated with primary antibody against Lamin A/C (Santa cruz biotechnology, inc.) with 50 fold dilution for 1 h, washed three times with PBS and then incubated with Alexa Fluor^® ^568 goat anti-mouse IgG (H+L) secondary antibody for 1 h (1: 100 dilution each). The slides were then washed three times with PBS, counterstained in Prolong^® ^Gold antifade reagent with DAPI and visualized with a BX41 system microscope (Olympus) or with an Olympus Fluoview confocal microscope using a 100× oil immersion objective lens (IX70 Olympus, Melville, NY, USA). Fluorescence was excited by the 488-nm line of an argon laser and the 568/647-nm line of a Krypton laser. Images were analyzed using Fluoview software.

### Senescence-associated β-galactosidase (SA β-Gal) staining

PC-3 cells with stable progerin-pEGFP, LA-pEGFP or pEGFP vector expression were seeded in six-well plates. When reaching 70% confluence, the cells were washed twice with PBS and fixed in fixation buffer containing 2% formaldehyde/0.2% glutaraldehyde in PBS for 7 min at room temperature. SA-β-gal staining was performed in fresh senescence-associated X-Gal staining solution containing 1 mg/ml of 5-bromo-4-chloro-3-indolyl beta-D-galactoside (X-Gal), pH 6.0, 5 mM potassium ferrocyanide, 5 mM potassium ferricyanide and 2 mM MgCl_2 _in PBS at 37°C (no CO_2_). Incubation typically lasted for 16 h. Cells were rinsed in PBS and stored in PBS with 70% glycerol. Cells were then examined under a microscope at Χ200 magnification for a blue-green staining of the cytoplasm indicative of senescence.

### Estimation of DNA damage by comet assay

We performed the comet assay on Comet Slide (Trevigen) following the manufacturer's instructions. Briefly, the cells were treated with camptothecin (0.1 μM) for 12 hours and the extent of DNA damage was measured 48 hours later. Dakin-frosted slides were covered with 100 ml of 0.6% normal melting point agarose, and the agarose was allowed to solidify under a cover slip on ice. The cover slips were then removed. Subsequently, aliquots (1 ml) of harvested cells containing 1 × 10^5 ^cells in culture medium were then centrifuged to pellets. The pellets were resuspended in 80 μl of 0.6% low melting point agarose, layered onto the normal melting point agarose and allowed to solidify under a fresh cover slip on ice. All the steps described were conducted under a reduced light level to prevent additional DNA damage. Slides were then placed immediately in a cold lysis buffer for 1 hour. After lysis, the slides were drained and placed in a horizontal gel electrophoresis tank, surrounded by ice and filled with fresh cold electrophoresis buffer to a level of ~0.25 cm above the slides. Slides were kept in the high pH buffer for 20 min, to allow DNA unwinding. Electrophoresis was then carried out for 20 min at 25 V and 300 mA. The slides were stained with SYBR Green and covered with a cover slip for immediate analysis. The slides were observed with a BX41 fluorescence microscope (Olympus, Center Valley, PA) and more than 100 cells were analyzed to give a representative result for the population of cells. The parameters of comet images such as the length of the tail moments were calculated using Comet Assay IV software (Perceptive Instruments Ltd).

### Cell viability measurement

Cell viability was measured with Vi-CELL™ Series Cell Viability Analyzers (Beckman Coulter, Inc.), which is based on traditional cell viability method of trypan blue exclusion.

### Animal model and histology

PC-3 (4 × 10^6^) cells with stable progerin-pEGFP, LA-pEGFP or pEGFP vector expression were injected subcutaneously into nude mice. Five groups of nude mice were utilized. Tumor development was monitored 1 week after implantation. About 38 days after implantation, the experiment was terminated and the animals were sacrificed. Primary tumors were removed and fixed for hematoxylin & eosin staining and immunohistochemical analysis. Mitotic and apoptotic figures in tumor sections stained with H&E were counted through the microscope by trained technicians with a mechanical tabulator, in a double blind approach. Total background cells in each field were also counted and the percentage of positive cells [(X positive/Y total count) × 100] was calculated for each tumor.

### Statistical analysis

The probability of statistically significant differences between two experimental groups was determined by Student's t-test. *P *< 0.05 was considered statistically significant in all calculations.

## Results

### Abnormal nuclear envelope proteins distribution in human cancer cell lines

**Figure 1:**
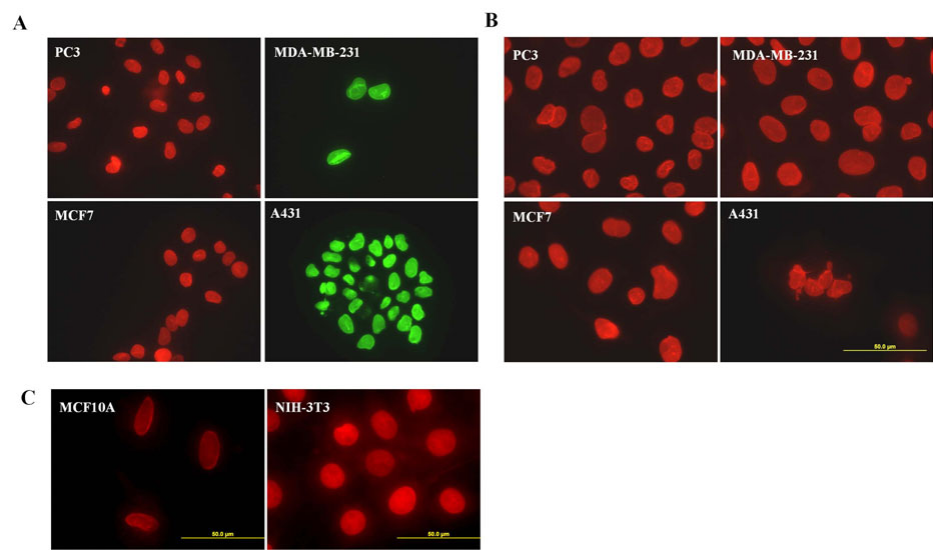
**Abnormal distribution of nuclear envelope proteins in human cancer cell lines**. Immunocytostaining of PC-3, MDA-MB-231, MCF7 and A431 with antibody against Lamin A (A) or emerin (B). Representative images of cells were taken with a fluorescence microscope at 400 × magnification (A) or 600 × magnification (B). Note the invaginations and blebbings pattern of Lamin A and emerin distribution. (C) Immunocytostaining of Lamin A in immortalized and non-malignant NIH 3T3 cells (Mouse embryonic fibroblast cell line) and MCF10A cells (human mammary epithelial cell-line). Representative images of cells were taken with a fluorescence microscope at 600 × magnification.

Nuclear envelope (NE) abnormality is an important diagnostic marker in a plethora of human cancers [12,13]. In sharp contrast to the nearly spherical shape of the NE in most normal cells, the NE of cancer cells predominantly displays irregularities characterized as passive distortion, increased chromatin aggregation, and deep infoldings. As shown in Figure 1a, immunocytostaining of lamin A revealed substantial nuclear lamina invaginations and blebbings in A431, MDA-MB-231, MCF7 and PC-3 cells, similar to those described in HGPS cells. In addition, emerin, which is an integral membrane protein and directly interacts with nuclear lamins, also displayed pronounced invaginations and blebbings distribution (Figure 1b).

### Existence of progerin in human cancer cell lines

**Figure 2:**
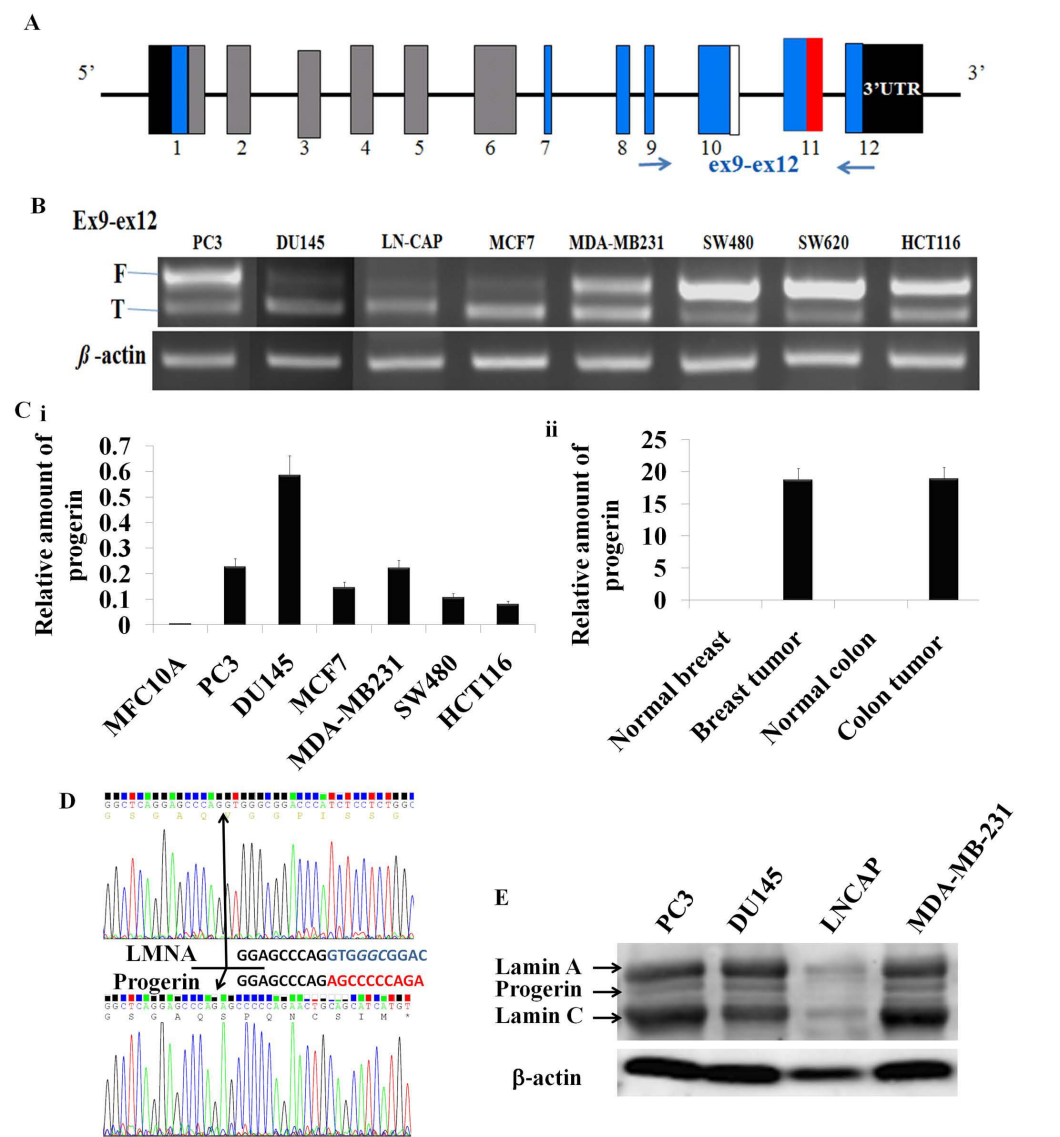
**Existence of progerin in human cancer cell lines**. (A) The 12-exon of Lamin A transcript is presented. The cryptic splice site (red box) and the position of the primer set used for RT-PCR is indicated. (B) RT-PCR analyses of progerin transcription in different cancer cells using primers detecting both the full-length (F) and the truncated (T) LMNA isoforms. All PCR products were confirmed by DNA sequencing. (C) Quantitative real time PCR analysis progerin or LMNA from a number of cancer cells (i), a human mammary epithelial cell-line (i), normal and tumor human tissues (ii). Δ150 LMNA RNA was detected by using Dex11-ex12 primers relative to total LMNA RNA detected by using ex8-ex9 primers is shown. (D) Sequencing of progerin and wildtype LMNA RT-PCR product from PC-3 cells. Progerin specific mRNA sequence (GGAGCCCAGAGCCCCCAGA) was compared with the normal *LMNA *sequence (GGAGCCCAGGTGGGCGGAC). (E) Western blotting of total cell lyses from PC-3, DU145, LNCAP, and MDAMB231 with Lamin A antibody, which detects lamin A/C and progerin simultaneously.

Most HGPS cases are caused by a nucleotide substitution at position 1824 (C to T) related 150 nucleotides deletion within *LMNA *mRNA [1]. We determined whether cancer cells also harbor mutant lamin A (progerin) using RT-PCR with primers that can simultaneously detect both the full-length (F) *LMNA *mRNA (510 bp) and the truncated (T) *LMNA *isoform/progerin (360 bp) (Figure 2a). The 510 and 360 bp fragments of the PCR products amplified from PC-3, DU145 and LNCaP cells were detected (Figure 2b).

Quantitative RT-PCR analysis using the splice junction primers demonstrated that use of the cryptic splice site (progerin) was about 38-fold higher in a number of cancer cell lines than in a normal human mammary epithelial cell-line (MCF10A) (Figure 2Ci). Use of the cryptic splice site is not because of cell-culture artifact, because Δ150 LMNA (progerin) mRNA is also detectable in human tissues (Figure 2Cii). Similar to cultured cells, we observed a 20-fold higher progerin mRNA level in human tumor tissues than it from normal human tissues (Figure 2Cii). In our study, the detection of progerin mRNA from human normal tissues, albeit at very low level compared with that from tumor tissue, is in agreement with a previous report that Δ50 lamin A (progerin) is present in cells from healthy individuals [10]. Sequence data confirmed the presence of expected lamin transcript fragments and mutation-related specific nucleotide fragments (Figure 2D). Further, western blotting was performed with primary antibody which simultaneously detects lamin A/C and progerin. An additional band is present between the lamin A and lamin C bands in all cell lines examined. The band is 3.8 kD lower than lamin A and is in consistent with the expected molecular weight of progerin (Figure 2E). Taken together, we conclude that progerin is expressed in a number of human cancer cell lines.

### Ectopic progerin expression in cancer cells

**Figure 3:**
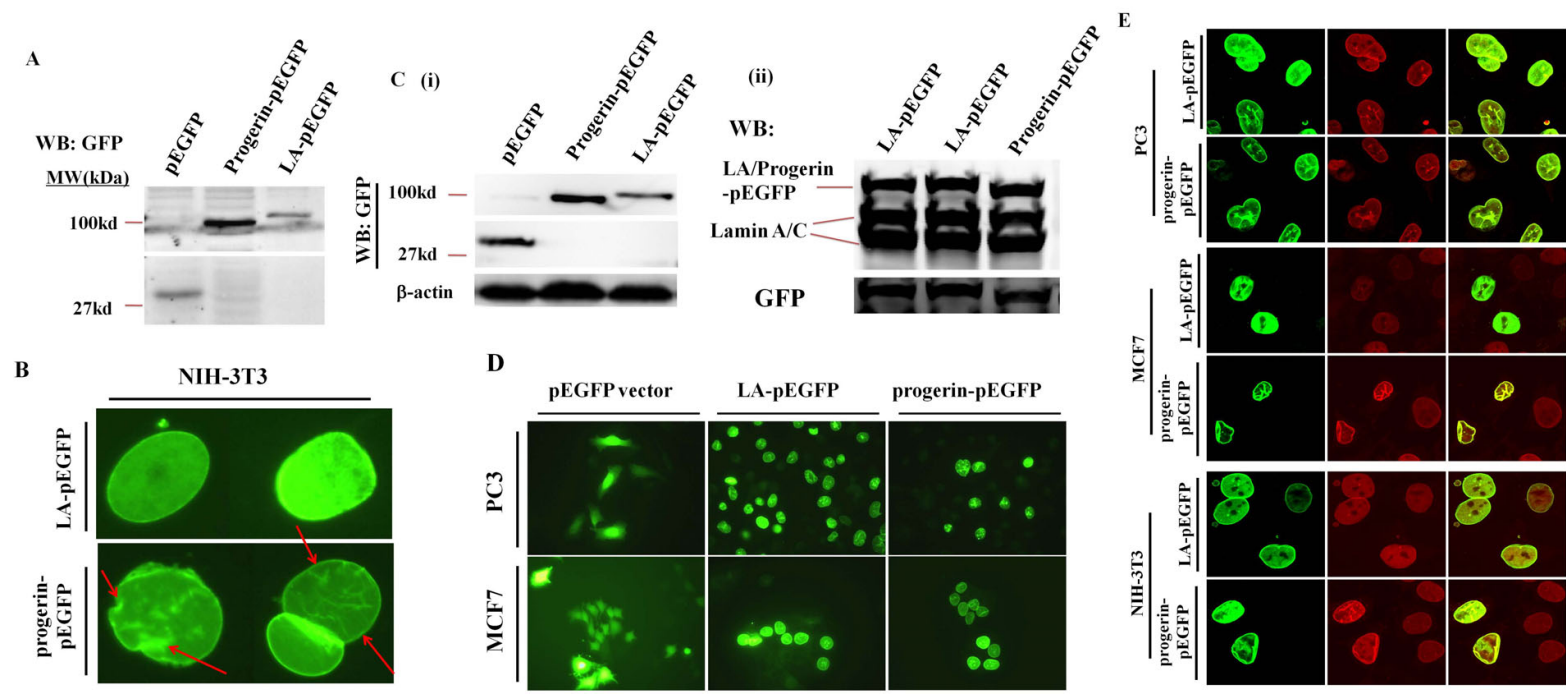
**Ectopic progerin expression in cancer cells**. NIH-3T3 cells were transfected with different lamin A expression constructs or the vector control. (A) Total cell lysates were probed with GFP antibody for Western blotting. (B) Representative images of cells were taken with a fluorescence microscope at 600 × magnification. Note that, ectopic expression of progerin exacerbated nuclear lamina distribution by inducing nuclear blebbings and invaginations (low panel), indicated by arrows. (C) PC-3 and MCF7 cells were transfected with lamin A expression constructs or vector control. Total cell lysates of PC-3 cells were probed with GFP antibody (i) or GFP and Lamin A/C antibody (ii) for Western blotting. (D) Representative images of cells were taken with a fluorescence microscope at 400 × magnification. (E) The PC-3, MCF7 and NIH-3T3 cells were transfected with Lamin A or progerin expression constructs. The cells were stained with Lamin A antibody. Representative images were obtained with an Olympus Fluoview confocal microscope using a 100× oil immersion objective lens.

To study the effects of progerin expression on cancer cells, PC-3 or MCF7 cells were transfected with wild type pEGFP-lamin A (LA-pEGFP), Δ50 pEGFP-lamin A (progerin-pEGFP) or a backbone vector. NIH-3T3 cells were also transformed. The expression of GPF fused progerin or wild type lamin A was confirmed with western blotting and immunocytostaining (Figure 3a, c and 3e). Ectopic progerin expression in NIH-3T3 cells resulted in blebbings, invaginations and cytoplasmic aggregates on the nuclear membrane, similar to those observed in HGPS fibroblasts (Figure 3b) [14,15]. In contrast, homogeneous and smooth distribution of LA-pEGFP was observed in cells transfected with the LA-pEGFP expression construct (Figure 3b). The results confirmed the detrimental effects of progerin on nuclear lamina distribution. Similarly, ectopic progerin expression in PC-3 or MCF7 cells was associated with prominently exacerbated intra-nuclear structures including nuclear invaginations and blebbings (Figure 3d and 3e). However, as different from the homogenous LA-pEGFP distribution in NIH-3T3 cells, LA-pEGFP displayed aberrant distribution in PC-3 and MCF7 cells (Figure 3d and 3e).

### Ectopically expressed progerin failed to induce cellular senescence in the cancer cells

**Figure 4:**
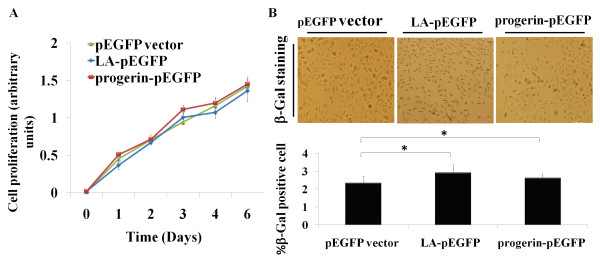
**Ectopically expressed progerin failed to induce cellular senescence in the cancer cells**. (A) The growth curve of PC-3 cells transfected with lamin A expression constructs or vector control was performed with a MTS assay. The bars represent the mean ± SE (n = 4). (B) SA-beta-Gal staining of PC-3 cells transfected with different lamin A expression constructs or vector control. Photomicrographs were taken at 200 × magnification. Percentages of SA-beta-Gal positive staining were presented below. *P < 0.01, compared with pEGFP vector transfected group.

Primary cultured cells can undergo cellular senescence *in vitro*, withdrawing from cell cycle progression. In contrast, malignant tumor cells can proliferate endlessly and rarely undergo cellular senescence *in vitro *[16]. A hallmark of HGPS fibroblasts and skin fibroblasts from a mouse model of progeria is their premature commitment to cellular senescence [14]. In this study, PC-3 cells transfected with either LA-pEGFP or progerin-pEGFP did not show marketed difference in proliferation (Figure 4a). Additionally, no noticeable difference regarding the cellular senescence, reflected by β-gal positively staining, was observed in PC-3 or MCF7 cells with ectopic progerin expression (Figure 4b). This finding indicates that the ectopic progerin expression failed to induce cellular senescence in PC-3 or MCF7 cells.

### Impaired DNA repair mechanisms in PC-3 cells with ectopic progerin expression

**Figure 5:**
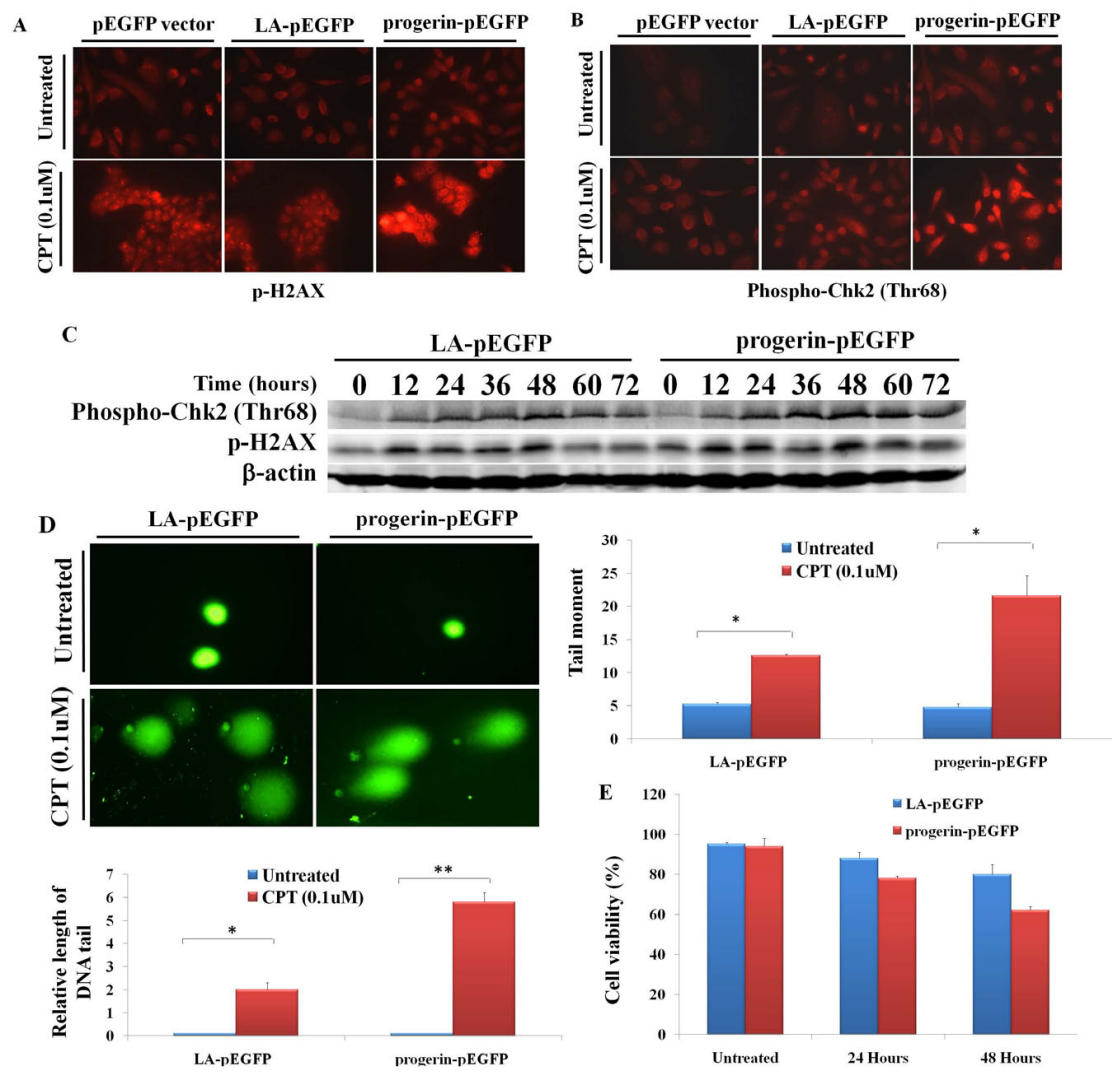
**Impaired DNA repairs in PC-3 cells with ectopic progerin expression**. PC-3 cells were transfected with different lamin A expression constructs or vector control. The cells were treated with CPT (0.1 μM) for 12 hours and 48 hours later, the cells were stained with either γ-H2AX (A) or Phospho-Chk2 (Thr68) (B) antibody. Representative images of cells were taken with a fluorescence microscope at 400 × magnification. (C) PC-3 cells were transfected with different either progerin-pEGFP or LA-pEGFP as control. The transfected cells were treated with CPT (0.1 μM) with the indicated times. The total cell lysates were probed with either γ-H2AX or Phospho-Chk2 (Thr68) antibody. (D) Representative photomicrographs of the comet assay showing the DNA migration pattern. Representative images of cells were taken with a fluorescence microscope at 600 × magnification. Relative lengths of the DNA tail and Tail moments are presented as below. The bars represent the mean ± SE (n = 20). *P < 0.01; ** P < 0.001, compared with PBS control at the corresponding time. (E) The cells were treated with CPT (0.5 μM) for 12 hours and 48 hours, the cell viability was measured by trypan blue exclusion based cell staining.

Progerin acts in a dominant negative manner to perturb DNA damage response and repair. The accumulation of progerin promote the cells' sensitivity to DNA-damaging agents, including ultraviolet irradiation and chemicals causing double-strand breaks (DSBs), which contributes to genomic instability in HGPS fibroblasts [7]. To determine whether progerin expression would perturb DNA repairs, we treated the cells with camptothecin, which generates double stranded DNA breaks (DSBs) by inhibiting eukaryotic DNA topoisomerase I [17]. Particularly, we focused on H2AX, which is phosphorylated (γ-H2AX) at the sites of double-stranded DNA breaks to initiate DNA damage checkpoint responses, thereby acting as the direct marker of DNA damage; and Chk2, which is an important component of the DNA damage checkpoint response [18]. Using immunofluorescence staining, we observed more γ-H2AX and Chk2 foci in PC-3 cells with ectopic progerin expression, indicating higher DNA damage in these cells (Figure 5a and 5b). Increased DNA damage was further confirmed by western blotting, which showed elevated phosphorylation of γ-H2AX and Chk2 at Thr68 (Figure 5c). Thus, PC-3 cells with progerin expression showed elevated sensitivities to the DNA-damaging agent, while the presence of phosphorylated γ-H2AX and Chk2 indicates compromised DNA damage repair mechanisms in those cells.

To determine whether the sustained checkpoint responses result from defective DNA repair, we applied alkaline comet assay to determine the DNA strand breaks [19]. The CPT treatment led to significant increase in the DNA damage in PC-3 cells with ectopic progerin expression as evidenced by elongated comet tail length and higher tail movement compared with those cells transfected with wild type lamin A (Figure 5d). The results indicate that the ectopic progerin expression compromises DNA repair in PC-3 cells. The delayed DNA damages repair response and the accumulation of impaired DNA in PC-3 cells with progerin expression potentiated the sensitivity of the cells toward CPT induced cell death (Figure 5e).

### Elevated tumorigeneis of PC-3 cells with ectopic progerin expression

**Figure 6:**
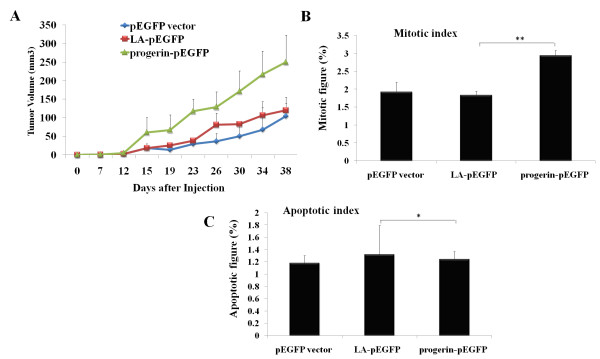
**Elevated tumorigeneis of PC-3 cells with ectopic progerin expression**. (A) Five-week growth kinetics of the tumors derived from PC-3 with stable Lamin A, progerin or vector expression. Data point, mean volume of 5 tumors for each group; Data point, mean volume of five tumors; bars, STD. (B) Mitotic index. Mitotic figures were counted in 400× fields and expressed as the percentage of the total cells. Columns, mean mitotic index in tumors; bars, STD. Note the significant increase in mitotic figures in tumors derived from progerin transfectants (n = 5) as compared to those of LA-pEGFP transfectants (n = 5) (p < 0.001 by Student's t test). (C) Apoptotic index. Apoptotic figures were counted in 400× fields and the index expressed as the percentage of the total cells in the same field. Columns, mean apoptotic index in tumors; bars, STD. (p < 0.01 by Student's t test).

Genomic instability is a step in multi-step tumorigenesis [8,9,20]. Genomic instability provides increased chances for the occurrence of DNA mutation related tumor suppressor genes malfunction or oncogenes hyperactivation, both of which are critical for tumor progression and growth [8,9,20]. To investigate whether a compromised DNA repair caused by ectopic progerin expression facilitates tumorigenesis of cancer cells *in vivo*, we injected PC-3 cells with the expression of LA-pEGFP, progerin-pEGFP or an empty plasmid pEGFP vector into nude mice, respectively. As shown in Figure 6a, tumors derived from PC-3 cells with progerin-pEGFP expression grew faster than those derived from LA-pEGFP or vector controls. Histological evaluation revealed an increase in mitotic index in tumors with progerin expression (Figure 6b). No statistically significant differences regarding the apoptotic index were found in tumors derived from progerin-transfected cells when compared to that of control tumors (Figure 6c). Taken together, the data suggest that progerin promotes the tumorigenic potential of PC-3 cells.

## Discussion

In the present study, the expression of progerin, a truncated form of lamin A protein, was detected in several human cancer cell lines. Ectopic expression of progerin in PC-3 and MCF7 cells compromised the DNA damage responses and repair, but did not induce cellular senescence. Importantly, an enhanced progerin expression conferred PC-3 cells with stronger tumorigenic potential *in vivo*. Together, these data showed that progerin, the origin of progeria disease (HGPS), may promote tumor genomic instability and contribute to tumorigenesis.

Progerin compromises the DNA damage repairs in HGPS fibroblasts. The defective response to DNA damages causes persistent activation of DNA damage responses proteins, such as γ-H2AX and p53-BP [4-7]. To clarify the effects of progerin expression in cancer cells, we introduced progerin and wildtype lamin A into prostate cancer PC-3, breast cancer MCF7 cells and mouse fibroblast NIH-3T3 cells. The nuclear envelope of NIH-3T3 progerin transfectants displayed blebbings, invaginations and cytoplasmic aggregates, similar to those in HGPS fibroblasts (Figure 3b), consistent with the dominant negative effects of progerin on nuclear lamina. When PC-3 or MCF7 cells were transfected with progerin, noticeable nuclear membrane invaginations were observed. Interestingly, the introduced wildtype lamin A existed at an invaginated pattern as well (Figure 3d and 3e). Divergent patterns of LA-pEGFP distribution in NIH-3T3 and cancer cell lines are probably due to the existence of endogenous progerin in cancer cells. Progerin only has a 50 C-terminal amino acid deletion from lamin A while retaining the central coiled-coil domain [21]. This indicates that exogenous LA-pEGFP may associate with endogenous progerin to form heterodimers through the coiled coil domains of the central rod region, thus reflecting the endogenous progerin distribution within the nuclear envelope. On the other hand, our results are in agreement with the observation that introduction of wildtype lamin A protein does not rescue the cellular symptoms within fibroblast from HGPS patients [15].

A hallmark of HGPS fibroblasts and skin fibroblasts from a mouse model of progeria is their premature cellular senescence [14]. The cellular senescence may be due to the increased DNA damages and corresponding cellular DNA damage responses [5-7]. In our study, we did not observe a significant difference regarding the proliferation rate of PC-3 cells expressing either progerin or vector control *in vitro *(Figure 4a). Introduced progerin failed to induce cellular senescence within PC-3 and MCF7 cells (Figure 4b). However, we observed increased DNA damages and persistent activation of DNA damage repair proteins, such as γ-H2AX and Chk2, in PC-3 progerin transfectants (Figure 5), when the cells were subjected to the DNA-damaging agent CTP. The results are consistent with earlier observations that progerin acts in a dominant fashion to trigger DNA repair defects [7,14,15]. A variety of factors involved in detecting and responding to DNA damage are altered in human tumors. Genotoxic stresses and genomic stabilities caused by defective DNA damage repair contribute to tumorigenesis [8,9,20]. In our study, tumors derived from PC-3 progerin transfectants grew much faster than those derived from control groups (Figure 6a). The increased growth rate is associated with higher mitotic index, while the apoptotic index is similar to that of control group (Figure 6b and 6c). Compared with those cells cultured *in vitro*, the *in vivo *transplanted cells are encountered with much more adverse environment, such as limitation of nutrient and oxygen supply primarily due to a poorly developed and/or malfunctioning vascular network, which might be associated with hypoxic stress, oxidative stress, increased DNA damages and genomic instability. Most of those factors would contribute to the enhanced tumorigenesis of the cancer cells.

Interestingly, a recent study by Marinõ et al. reported that an extensive basal activation of autophagy occurs in Zmpste24-null mice, which show accelerated aging and is a reliable model of human Hutchinson-Gilford progeria [22]. Autophagy may restrain tumorigenesis by maintaining genome stability, which is pivotal for coping with metabolic stress encountered by established aggressive tumors [23]. It is believed that nuclear abnormalities causing premature aging in Zmpste242/2 mice trigger a metabolic response involving the activation of autophagy [22]. Nevertheless, the underlying induction and regulation mechanisms responsible for the elevated autophagy flux in progeria mouse model await further investigation. The study might partially explain the progerin promoted tumorigeneis of PC-3 cells in our study.

To elucidate whether ectopic progerin expression could render the non-malignant/transformed cells with tumorigenesis ability, we transfected NIH-3T3 cells with either LA-pEGFP or progerin-pEGFP and tried to establish NIH-3T3 sublines that stably express those proteins. However, the efforts are hampered by the growth disadvantages presented by the NIH-3T3 cells with ectopic progerin-pEGFP expression. Meanwhile, we think progerin promoted tumorigenesis is based on its ability to compromise functional DNA-damage repair, which is important to maintain the genomic integrity when the tumors reach the size limits where suppressed nutrient and oxygen supply contributes to the hypoxic and oxidative stress. In another word, progerin expression would not promote the tumorigeneis at its very initial stage of tumor development.

In summary, here we report for the first time that progerin, a mutated lamin A that occurs in a rare genetic disorder characterized by premature senescence, is expressed in a variety of established human cancer cell lines. Although PC-3 cells transfected with progerin did not show any phenotype of cellular senescence, there were increased DNA damages and persistence of DNA damage responses in the progerin transfectants after CPT treatment. Progerin transfectants displayed increased tumorigenesis ability *in vivo*, probably due to heightened genomic instabilities. Finally, given the efficiency of farnesyltransferase (FTase) inhibitors (FTIs) in the nuclear architecture rescue of human HGPS fibroblasts and a broad spectrum of human cancer treatments, the targeting of progerin may open a new avenue for human cancer therapy [24-27].

## Abbreviations used

HGPS: Hutchinson-Gilford progeria syndrome; ATM: ataxia telangiectasia mutated; ATR: ataxia telangiectasia mutated and rad3-related.

## Conflict of interests

The authors declare that they have no competing interests.

## Authors' contributions

YT and DN conceived the project and designed the experiments. YT, YC and HJi performed the studies. YT prepared and DN edited the manuscript. DN gave approval for the final version to be submitted.
